# *Salmonella* spp. in Serbian poultry production (2020–2025): Retrospective trends and farmers’ perceptions of biosecurity

**DOI:** 10.1002/vro2.70049

**Published:** 2026-09-14

**Authors:** Jelena Maletić, Nemanja Zdravković, Jasna Kureljušić, Branislav Kureljušić, Marco De Nardi, Oliver Radanović, Nemanja Jezimirović, Giulia Graziosi

**Affiliations:** ^1^ Scientific Institute of Veterinary Medicine of Serbia Belgrade Serbia; ^2^ Department of Veterinary Medical Sciences University of Bologna Ozzano dell'Emilia Italy

## Abstract

**Background:**

*Salmonella enterica* subsp. *enterica* remains a major zoonotic threat worldwide, with poultry products being a major source of human foodborne infections.

**Methods:**

A retrospective analysis of *Salmonella* spp. occurrence in Serbian poultry production was conducted using routine diagnostic data collected between 2020 and 2025. A total of 2453 samples from poultry farms, 147 from hatcheries and 3237 from broiler slaughterhouses were analysed. To complement the epidemiological investigation, a questionnaire was administered to 29 poultry farmers to assess difficulty associated with implementing measures from different biosecurity categories (BCs) and awareness of the economic consequences associated with *Salmonella* detection.

**Results:**

*Salmonella* spp. was detected in 2.9% of farm samples and 8.4% of slaughterhouse samples, whereas all hatchery samples tested negative. Across poultry categories, broilers showed the highest positivity rate (57/977, 5.8%), followed by layers (10/588, 1.7%) and breeders (4/741, 0.5%). Serotyping was available for a subset of farm isolates, with *S*. Enteritidis representing the most frequent serovar (83.3%). Generalised linear mixed‐effects models identified seasonality as a significant predictor of *Salmonella* occurrence. ‘Depopulation process’, ‘manure and carcass removal’, ‘biological vector control’ and ‘disease management’ were perceived as most challenging BCs.

**Conclusion:**

This study provides the first integrated assessment of *Salmonella* occurrence across multiple components of the Serbian poultry production chain, combining retrospective surveillance data with a farmer‐based evaluation of biosecurity implementation. The findings identify critical points for *Salmonella* transmission and provide evidence to support targeted, context‐specific biosecurity interventions.

## INTRODUCTION

Despite ongoing control efforts, salmonellosis remains one of the most common food‐borne zoonoses worldwide and a leading cause of gastroenteritis in humans.^1^, ^2^, ^3^, ^4^ Among non‐typhoidal *Salmonella* spp. (NTS), the serovars *S*. Typhimurium and *S*. Enteritidis are responsible for most human infections worldwide, followed by *S*. Infantis, *S*. Newport, *S*. Dublin, *S*. Heidelberg and *S*. Weltevreden.^5^, ^6^ Non‐typhoidal *Salmonella* spp. is primarily transmitted through the consumption of undercooked or contaminated animal‐derived food products or through direct contact with infected animals.^7^ Poultry populations, especially chickens and turkeys, are frequently infected with *NTS* without detectable symptoms.^8^ In Europe, where salmonellosis ranks as the second most common zoonosis,^1^ the prevalence of *Salmonella* in poultry flocks was 2.3% in 2023, with 0.58% of flocks testing positive for one of the five serovars targeted by national control programmes (*S*. Enteritidis, *S*. Typhimurium, including monophasic *S*. Typhimurium, *S*. Infantis, *S*. Virchow and *S*. Hadar), showing little variation compared with previous years.^1^ In Serbia, human disease burden of salmonellosis remains concerning, with 1217 confirmed cases in 2023.^9^ Most infections were linked to the consumption of contaminated poultry products, with *S*. Enteritidis, *S*. Infantis and *S*. Typhimurium being the most frequently identified serovars.^10^, ^11^, ^12^ Measures for the surveillance and control of public‐health‐relevant *Salmonella* serovars in broilers, laying hens and turkeys are established in national regulations.^13^ In accordance with the Serbian national control programme, detection of *S*. Enteritidis or *S*. Typhimurium in a flock requires stamping out or slaughter under strict transport precautions, with mandatory heat treatment of meat.^13^ According to the Serbian Regulation on general and specific food hygiene conditions and microbiological criteria for food,^14^ *Salmonella* must be absent from poultry carcasses, fresh poultry meat, meat preparations, eggs and egg products. Following a positive *Salmonella* detection, affected products are destroyed or recalled if they have already entered the market.^14^

Efforts to reduce salmonellosis in humans include the implementation of preventive and control measures throughout the poultry production chain, alongside consumer education and awareness programmes.^15^, ^16^ Key biosecurity measures (BMs) for *Salmonella* control on farms include the use of *Salmonella*‐free chicks, implementation of all‐in/all‐out production systems, prevention of contact with rodents and wild animals, provision of uncontaminated feed and water, and thorough cleaning and disinfection between production cycles.^16^, ^17^, ^18^ However, the implementation of BMs on farms appears to depend not only on economic constraints or practical feasibility, but also on farmers’ understanding of biosecurity principles and their attitudes towards and motivation for adopting disease‐prevention practices.^12^, ^19^, ^20^, ^21^, ^22^, ^23^ Further along the production chain, inadequate hygiene practices and cross‐contamination in slaughterhouses may facilitate the spread of *Salmonella* from infected flocks to poultry carcasses and meat products.^8^

Given the relevance of *Salmonella* from a One Health perspective, this study aimed to characterise its occurrence in primary production (farms and hatcheries) and processing (broiler slaughterhouses) in Serbia between 2020 and 2025 and to assess farmers’ perceptions of biosecurity implementation and awareness of the economic consequences associated with *Salmonella* detection.

## MATERIALS AND METHODS

### Study design and microbiological analysis

The occurrence of *Salmonella* in Serbian poultry production was investigated through a retrospective observational study encompassing primary production (commercial poultry farms and hatcheries) and processing (broiler slaughterhouses). Samples submitted for *Salmonella* spp. testing between January 2020 and June 2025 were collected from 49 commercial poultry farms, two hatcheries and 10 broiler slaughterhouses. The study period was selected to provide a recent overview of *Salmonella* occurrence within the Serbian poultry sector in the study area. All samples originated from routine diagnostic submissions to the Scientific Institute of Veterinary Medicine of Serbia (https://nivs.rs/?lang), triggered by clinical suspicion, regulatory monitoring or routine screening activities. The poultry farms and hatcheries were located in the Belgrade city epizootiological area. Slaughterhouse samples represented poultry sourced from multiple Serbian production areas; specific source‐region identifiers were not available in the anonymised retrospective dataset. Sampling locations reflected the availability of routine diagnostic submissions rather than prospective site selection. The complete anonymised dataset is provided in Supporting Information S1.

Sampling at poultry farms and hatcheries was carried out in accordance with national regulations governing the *Salmonella* surveillance programme in poultry.^13^ Sampling procedures varied according to poultry category and sampling context. Poultry‐farm samples consisted of a pair of tissue boot swabs (Finnimport) and/or faecal samples collected according to category‐specific surveillance protocols. Hatchery samples included incubated eggs, unhatched eggs at the end of the hatching process and hatched eggshells. In broiler slaughterhouses, neck‐skin samples, meat samples and environmental swabs from food‐contact surfaces (including working tables, knives, cutting boards and equipment in contact with raw poultry meat) were collected in accordance with national food hygiene and microbiological‐monitoring regulations.^14^

All samples were processed according to the EN ISO6579‐1:2017/A1:2020,^24^ with minor adjustments related to sample type. During pre‐enrichment, boot swabs and faecal material (25 g) were homogenised in 225 mL of buffered peptone water (BPW; Torlak Institut, Belgrade, Serbia) and incubated at 37 ± 1°C for 18 ± 2 h. Hatchery samples (eggshells and unhatched eggs) were aseptically processed to obtain 25 g of material, followed by homogenisation in 225 mL of BPW and incubation as described above. Slaughterhouse samples (meat and neck skin; 25 g per sample) and environmental swabs taken from food‐contact surfaces were processed in the manner using BPW pre‐enrichment.

Thereafter, three drops (33 µL each) of incubated BPW were inoculated onto a single plate of modified semisolid Rappaport‐Vassiliadis (HiMedia) medium, and plates were incubated for 24 ± 3 h at 41.5 ± 1°C. Samples without visible growth were incubated for an additional 24 h. Samples were streaked onto two selective media (xylose lysine deoxycholate and MacConkey agars; both HiMedia) and incubated for 24 ± 3 h at 37 ± 1°C. Colonies suspected to be *Salmonella* were confirmed biochemically (indole reaction, triple sugar iron agar, L‐lysine decarboxylation medium and urea agar) and, where available, serotyped according to ISO/TR 6579‐3:2014^25^ and the Kauffmann–White–Le Minor scheme.^26^

### Statistical analysis

To investigate which explanatory variables were associated with the probability of *Salmonella* detection in live poultry and slaughterhouse samples, logistic mixed‐effects regression models (GLMMs) with binomial distribution were applied.

Data visualisation and statistical analyses were conducted using R software (version 4.3.1). Before data analysis, each sampled facility was assigned an anonymised alphanumeric identifier (A to Z; AA to ZM). *Salmonella* positivity was calculated as the proportion of positive samples among those tested. When no positive samples were observed, 95% confidence intervals were computed using the exact binomial (Clopper‒Pearson) method. The binary outcome was *Salmonella* spp. presence (0 = absence; 1 = presence), with fixed and random effects selected according to the dataset. The ‘glmmTMB’ (version 1.1.14) and ‘lme4’ (version 2.0‐1) packages were used. Model selection followed a backward approach based on Akaike's information criterion (AIC), and the most parsimonious model was selected. For farm samples, ‘poultry type’, ‘season’ and ‘year’ were initially considered as predictors, with ‘farm’ included as a random intercept to account for repeated measures. Each predictor was tested separately and then in a multivariable model. For slaughterhouse samples, ‘type of sample’ and ‘season’ were initially considered as predictors, with ‘slaughterhouse’ included as a random intercept. Odds ratios (ORs) were calculated from model coefficients to quantify the strength of associations. ‘Broilers’ and ‘winter’ were the reference categories for ‘poultry type’ and ‘season’, respectively, because they had the highest observed *Salmonella* positivity in the farm dataset. Statistical significance was assessed for each predictor, and results were expressed as relative increases or decreases in the odds of positivity compared with the reference categories. Model adequacy was evaluated using the ‘DHARMa’ package through simulation‐based residual diagnostics, including tests for dispersion, zero inflation and uniformity of residuals. The R code and model diagnostics are provided in Supporting Information 2 (Figures S1–S6).

### Farm‐based questionnaire

A cross‐sectional survey was conducted to evaluate farmers’ perceptions of BMs implementation, identify major constraints to implementation, and assess their awareness of the economic impact of *Salmonella*‐control measures.

The survey was conducted in accordance with Regulation (EU) 2016/679 (General Data Protection Regulation) and the University of Bologna guidelines for the protection of personal data in surveys. In accordance with institutional guidelines for minimal‐risk, non‐interventional research involving adult participants and non‐sensitive data, formal ethical approval was not required. Participation was voluntary, and informed consent was obtained from all participants before completion of the survey.

The questionnaire comprised three sections. The first collected information on farm characteristics and previous *Salmonella* detections. The second assessed farmers’ perceptions of the difficulty associated with implementing measures from different biosecurity categories (BCs) in daily practice, using a five‐point Likert scale (very easy = 1, easy = 2, normal = 3, difficult = 4, very difficult = 5). An open‐ended question on the most problematic BC to implement was also included. Biosecurity categories were selected considering internal and external BMs from the Biocheck.UGent scoring systems for broiler and layer farms.^27^, ^28^ The following categories were considered: ‘access control (vehicles and visitors)’; ‘farm‐entry measures’ (e.g., changing clothes and boots); ‘cleaning and disinfection’; ‘biological vector control’; ‘manure and carcass removal’; ‘disease management’ (e.g., vaccination, treatment administration and sampling activities); ‘depopulation process’; ‘measures between poultry houses’ (e.g., changing or disinfecting footwear, using house‐specific equipment, and cleaning hands before moving from one house to another); and ‘working‐line practices’ (e.g., visiting younger flocks before older flocks on farms with birds of different ages). The third section evaluated farmers’ awareness of the economic consequences of detecting specific *Salmonella* serotypes under Serbian national legislation.^13^

The questionnaire was administered to poultry farmers randomly selected from the list of registered poultry holdings in the Belgrade city epizootiological area. Eligible participants were selected by simple random sampling (https://randomnumbergenerator.org/); non‐responders were replaced by the next randomly selected eligible participant. A total of 29 farms were ultimately enrolled, and the survey was administered during farm visits in October 2025.

Questionnaire data were compiled in a Microsoft Excel spreadsheet (version 16.103). Descriptive statistics were used to assess the perceived difficulty of implementing the different BCs and to identify those considered the most and least challenging. To explore similarities and differences between farms, responses were visualised using a heatmap based on hierarchical clustering,^29^ generated with the ‘pHeatmap’ package (version 1.0.12) in R (version 4.3.1). Because responses were collected on a five‐point Likert scale (ordinal data), clustering was performed using Gower distance. Hierarchical clustering iteratively groups observations according to their similarity, allowing the identification of clusters of farms with comparable perceptions and groups of BCs perceived as more or less difficult to implement.

## RESULTS

### *Salmonella* spp. detection across the poultry production chain

During the study period, a total of 2453 samples from poultry farms, 147 from hatcheries and 3237 from broiler slaughterhouses were submitted for microbiological analysis. Each facility was sampled between one and 12 times. A total of 1329 faecal samples (57.6%) and 977 boot‐swab samples (42.4%) were analysed. Of these, 977 samples originated from broilers (42.3%), 588 from layers (25.5%) and 741 from breeder flocks (32.1%). *Salmonella* spp. was isolated from 71 farm samples (2.9%) and 273 slaughterhouse samples (8.4%) (Table 1). All hatchery samples tested negative (0/147; 0%, 95% confidence interval [CI]: 0%‒2.5%). Farm positivity was highest in 2022 (10.3%), whereas no *Salmonella* spp. was detected in 2023 or 2024. Across poultry categories, detections were most frequent in broilers (57/977, 5.8%), followed by layers (10/588, 1.7%) and breeders (4/741, 0.5%). Serotyping was performed for a subset of *Salmonella*‐positive farm isolates (36/71), selected according to isolate availability and routine laboratory workflow at diagnosis. Slaughterhouse isolates were not serotyped. Four *Salmonella* serovars were identified; *S*. Enteritidis was the most frequent (30/36, 83.3%). It was isolated from all poultry categories, with most detections occurring in broilers (24/36, 66.7%). *S*. Typhimurium (3/36, 8.3%) and *S*. Infantis (2/36, 5.5%) were found only in broilers, whereas *S*. Mbandaka (1/36, 2.8%) was detected in layers.

**TABLE 1:** *Salmonella* spp. positivity in samples from poultry farms, hatcheries and broiler slaughterhouses from January 2020 to June 2025.

	2020	2021	2022	2023	2024	2025	Total
Samples from poultry farms and hatcheries, positive (%)	729, 18 (2.5%)	594, 12 (2%)	360, 37 (10.3%)	245, 0 (0; 95% CI: 0‒1.4)	358, 0 (0; 95% CI: 0‒1)	167, 4 (2.4%)	2453, 71 (2.9%)
Samples from broiler slaughterhouses, positive (%)	279, 20 (7.2%)	418, 63 (15.1%)	374, 7 (1.9%)	628, 70 (11.1%)	1070, 69 (6.4%)	412, 44 (10.7%)	3237, 273 (8.4%)

*Note*: For years with zero detections, exact binomial 95% confidence intervals (95% CIs) are shown in brackets.

Among broiler slaughterhouse samples, 2945 were meat samples (91.0%), 245 were environmental swabs (7.6%) and 47 were neck‐skin samples (1.5%). The highest annual *Salmonella* positivity occurred in 2021 (15.1%), whereas the lowest occurred in 2022 (1.9%). Slaughterhouse isolates were not serotyped; therefore, positive results refer to *Salmonella* spp. only.

**FIGURE 1:**
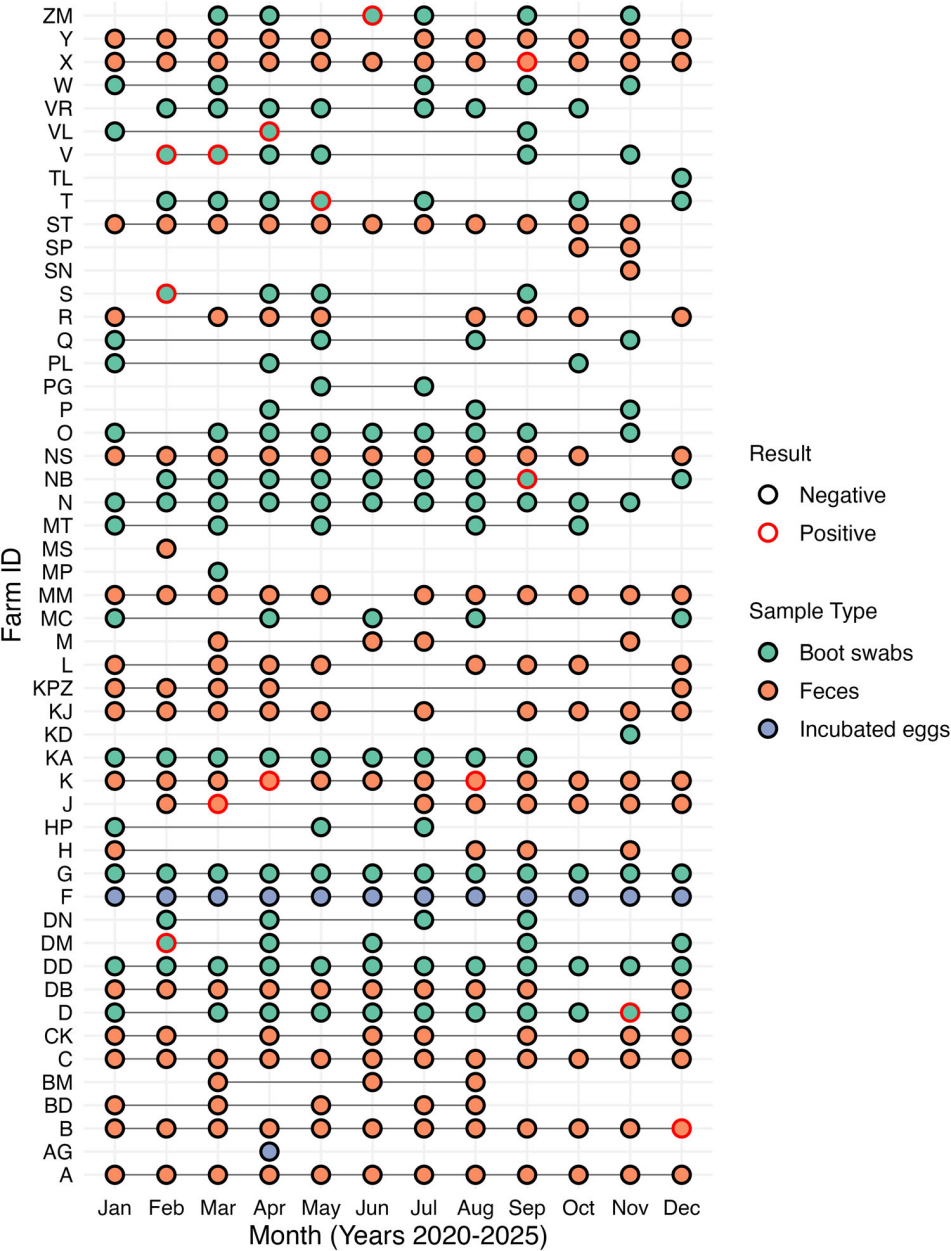
Temporal distribution of *Salmonella* spp. sampling and detections in 49 poultry farms and two hatcheries. Rows represent facilities identified by anonymised alphanumeric codes, and columns represent sampling months. Filled symbols indicate *Salmonella*‐negative samples, red‐outlined symbols indicate *Salmonella*‐positive samples, and colours denote poultry categories (broilers, layers and breeders).

**FIGURE 2:**
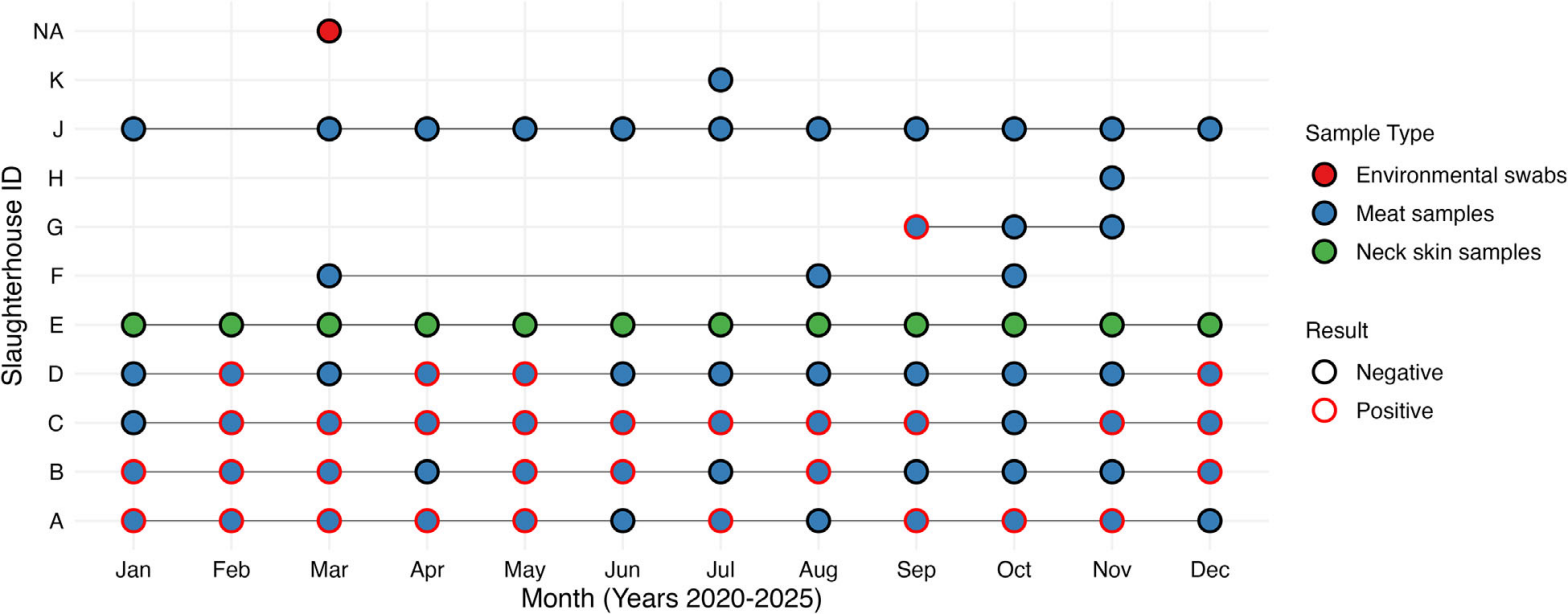
Temporal distribution of *Salmonella* spp. sampling and detections in 10 broiler slaughterhouses. Rows represent slaughterhouses identified by anonymised codes, and columns represent sampling months. Symbol shapes indicate sample type, and red‐outlined symbols indicate *Salmonella*‐positive samples.

Considerable variability in *Salmonella* positivity was observed among facilities. Farms V and K had positive results in more than one sampling month, whereas the other positive farms had detections confined to a single month or remained negative throughout the study (Figure 1). In slaughterhouses, *Salmonella* spp. was detected only in meat samples; repeated positive results over multiple months were recorded in slaughterhouses A, B, C and D, while samples from slaughterhouse G tested positive once (Figure 2).

**FIGURE 3:**
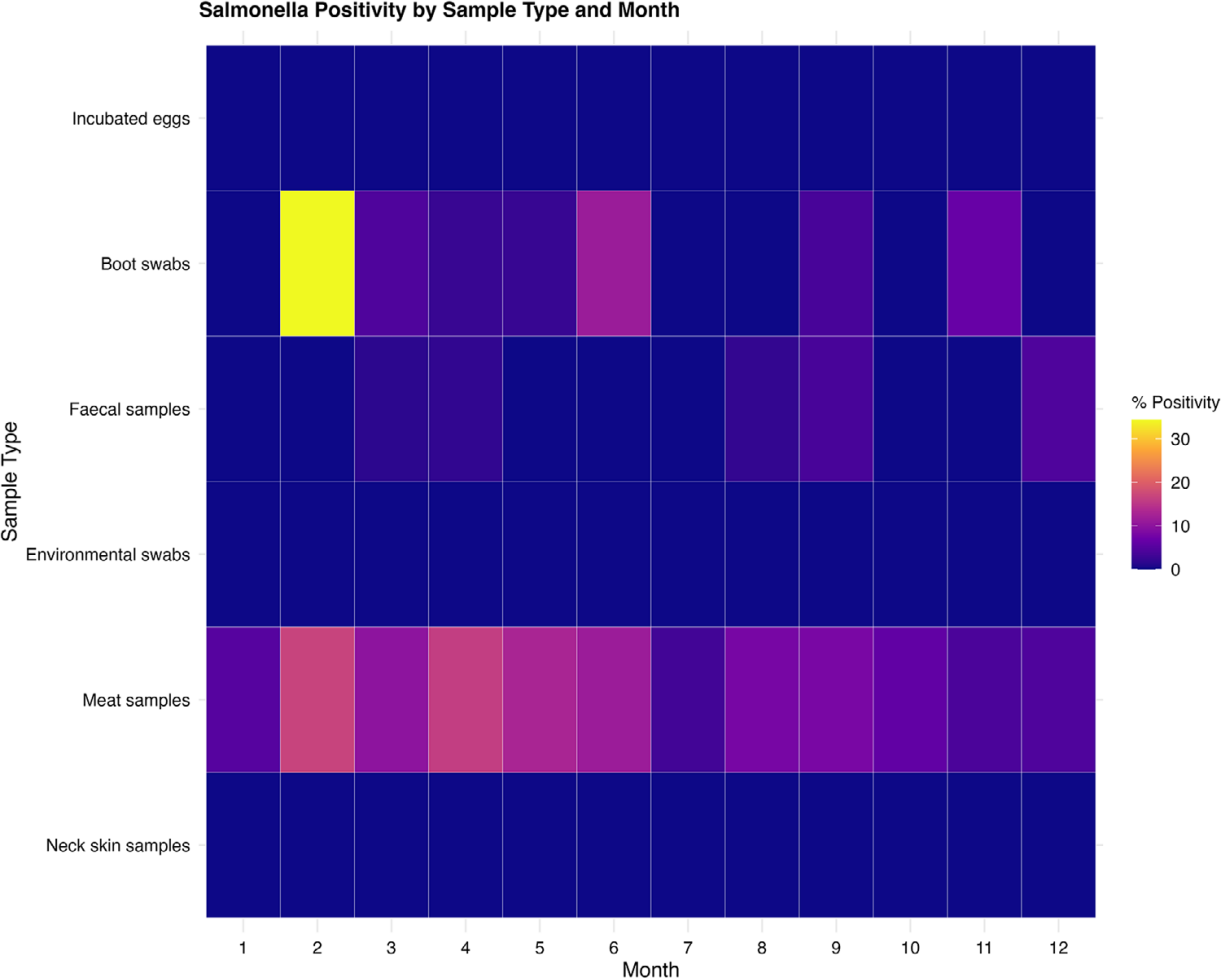
Monthly *Salmonella* positivity by sample type during 2020‒2025. Colour intensity represents the percentage of *Salmonella*‐positive samples in each sample category and month; warmer colours indicate higher positivity.

*Salmonella* positivity varied markedly by sample type and month (Figure 3). Boot swabs had the highest detection rates, with a peak in February (>30%) and smaller increases in April and later months. Meat samples collected in slaughterhouses showed moderate positivity (approximately 10%–20%) across several months. In contrast, incubated eggs, faecal samples, environmental swabs and neck‐skin samples were negative or rarely positive, without an apparent seasonal pattern.

### Statistical analysis

Results of the GLMMs are shown in Table 2. The final GLMM for the farm dataset included ‘poultry type’ and ‘season’ as fixed effects, with ‘farm ID’ and ‘year’ as random intercepts (AIC = 385.4). The random effect for ‘farm ID’ showed substantial between‐farm variability in baseline *Salmonella* positivity (*σ* ^2^ = 19.98, *σ* = 4.47), whereas year‐to‐year variability was modest (*σ* ^2^ = 1.74, *σ* = 1.32). ‘Season’ was the only significant predictor, with lower odds of *Salmonella* detection in spring (OR = 0.20, 95% CI: 0.09–0.46, *p* < 0.001) and summer (OR ≈ 0.30, 95% CI: 0.08–1.06, *p* = 0.042) compared to winter (the reference category). ‘Poultry type’ was not statistically significant, although layers and breeder flocks had lower point estimates than broilers (the reference category).

**TABLE 2:** Selected generalised linear mixed‐effects models and explanatory variables.

Dataset tested	Explanatory variable and levels		Estimate (standard error)	OR (95% CI)	*p‐*Value
Farm samples (AIC = 385.4)^a^	Poultry type^b^	Layers	‒2.09 (1.61)	0.12 (0.01‒11.3)	n.s.
		Breeders	‒2.71 (3.13)	0.07 (<0.01‒20.5)	n.s.
	Season^c^	Spring	‒1.60 (0.44)	0.20 (0.09‒0.46)	<0.001^***^
		Summer	‒1.21 (0.60)	0.30 (0.08‒1.06)	<0.05^*^
		Autumn	‒0.30 (0.52)	0.74 (0.30‒1.92)	n.s.
Slaughterhouse samples (AIC = 1768.2)^d^	Season^c^	Spring	0.54 (0.16)	1.72 (1.25–2.36)	<0.001^***^
		Summer	‒0.042 (0.20)	0.96 (0.64–1.46)	n.s.
		Autumn	‒0.417 (0.22)	0.66 (0.43–1.00)	n.s.

*Note*: Estimates (*β*), odds ratios (OR = exp(*β*)), 95% confidence intervals (CIs) and *p*‐values from *z*‐tests comparing each category with the reference category are shown.

Abbreviations: AIC, Akaike's information criterion; n.s., not significant; SD, standard deviation.

^a^ Variance and SD for the farm random effect: 19.98 and 4.47, respectively; variance and SD for the year random effect: 1.744 and 1.321, respectively.

^b^ Broilers were the reference category.

^c^ Winter was the reference category.

^d^ Variance and SD for the slaughterhouse random effect: 0.92 and 0.96, respectively.

* *p* ≤ 0.05.

*** *p* ≤ 0.001.

For slaughterhouses, only meat samples were included in the analysis because neck‐skin samples and environmental swabs yielded zero or near‐zero positive results. The final model included ‘season’ as the only fixed effect and ‘slaughterhouse ID’ as random effect (AIC = 1768.2). The variance component was moderate (*σ* ^2^ = 0.92, *σ* = 0.96). *Salmonella* positivity was significantly higher in spring than in winter (OR = 1.72, 95% CI: 1.25‒2.36, *p* < 0.001), whereas summer and autumn did not differ significantly from winter.

Model diagnostics confirmed adequate fit for both GLMMs, with no evidence of overdispersion, zero inflation or bias in residuals.

### Farm‐based questionnaire

Responses on the perceived difficulty of implementing measures belonging to different BCs in daily farm operations are summarised in Table 3. Among farms, 58.6% reared broilers, 27.6% layers and 13.8% breeders. *Salmonella* had been confirmed at least once on 37.9% of the surveyed farms. Most farms (72.4%) had more than 5000 birds, 24.1% had 1001–5000 birds and 3.4% had 500–1000 birds. In the open‐ended question, ‘depopulation process’ was identified as the most challenging BC (15 mentions, 51.7%), followed by ‘cleaning and disinfection’ (10, 34.5%), ‘manure and carcass removal’ (10, 34.5%) and ‘measures between poultry houses’ (8, 27.6%). Most respondents (27/29, 93.1%) were fully aware of the economic consequences of detecting certain *Salmonella* serotypes; one was partially aware (3.4%) and one was unaware (3.4%). Reported economic impacts included reduced production, additional testing costs, bans on egg or meat sales, and cleaning and disinfection expenses.

**TABLE 3:** Farmers' perceived difficulty in implementing measures within different biosecurity categories (BCs).

BCs	*N*	Min.	Max.	Average
Access control (vehicles and visitors)	29	1	4	1.97
Farm‐entry measures	29	1	3	2.20
Cleaning and disinfection	29	1	4	2.62
Biological vector control	29	1	4	2.69
Manure and carcass removal	29	1	4	3.41
Disease management	29	1	4	2.86
Depopulation process	29	2	4	3.31
Measures between poultry houses	29	1	4	2.83
Working line practices	29	1	4	1.21

*Note*: A five‐point Likert scale was used (very easy = 1, easy = 2, normal = 3, difficult = 4, very difficult = 5). The number of respondents (*N*), minimum (Min.), maximum (Max.) and mean score are shown.

**FIGURE 4:**
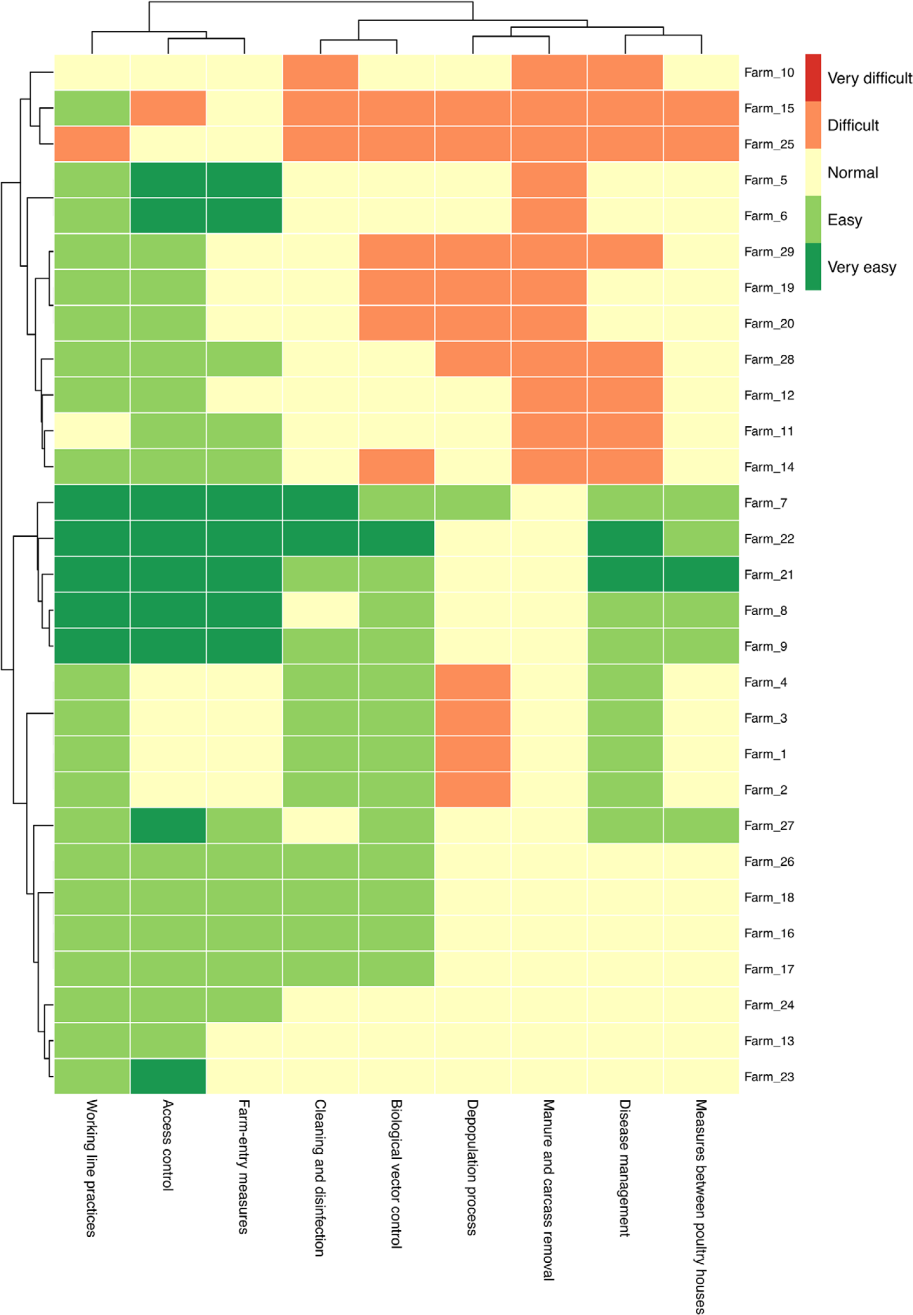
Heatmap of farmers' perceived difficulty in implementing measures of different biosecurity categories (BCs) across surveyed poultry farms. Rows represent farms and columns represent the nine BCs assessed. Colours show Likert scores from 1 (very easy) to 5 (very difficult), with green indicating lower and red indicating higher perceived difficulty. Farms were grouped by hierarchical clustering using Gower distance.

Hierarchical clustering of ordinal responses for the nine BCs assessed across 29 poultry farms revealed distinct patterns in perceived implementation difficulty (Figure 4). Most farms reported easy‐to‐normal scores for most BCs, suggesting familiarity with basic biosecurity practices. A smaller cluster reported several measures as difficult or very difficult. ‘Manure and carcass removal’ and ‘depopulation process’ tended to cluster and were more often scored as difficult or very difficult. ‘Biological vector control’ and ‘disease management’ were also commonly rated as difficult, although with greater between‐farm variability. In contrast, ‘access control’, ‘farm‐entry measures’ and ‘cleaning and disinfection’ were generally rated as easier to implement, with lower and more consistent scores.

## DISCUSSION

This study presents a retrospective analysis of *Salmonella* spp. occurrence on poultry farms, hatcheries and in broiler slaughterhouses in Serbia between 2020 and 2025, based on diagnostic samples submitted to the Scientific Institute of Veterinary Medicine of Serbia, Belgrade. Overall *Salmonella* spp. positivity was 2.9% in live poultry and 8.4% in broiler slaughterhouse samples.

During the study period, annual farm positivity varied, with the highest value in 2022 (10.3%) and no detections in 2023 a 2024. Because submissions arose from clinical suspicion, regulatory monitoring and routine screening rather than a fixed probability‐based sampling frame, the temporal pattern may be affected by selection bias arising from changes in submission intensity, farm participation or the reasons for testing. The findings should therefore be interpreted as patterns in diagnostic submissions rather than unbiased estimates of temporal prevalence. Although only a subset of farm isolates was serotyped, *S*. Enteritidis was most frequent, particularly in broilers, consistent with previous studies in Serbia, including the Belgrade, South Bačka and Srem areas.^11^, ^30^ This appears to contrast with the broader European epidemiological situation during the same period, in which *S*. Infantis was among the most frequently isolated serovar in poultry and has predominated in broiler production in Member States including Italy, Hungary and Poland since 2014.^1^, ^31^, ^32^, ^33^

Only a limited number of poultry holdings had repeated *Salmonella* spp. detections; most remained negative or yielded positive results only once. *Salmonella* introduction therefore appeared largely episodic, although a small number of holdings may have harboured recurrent infection sources or persistent environmental contamination.^34^, ^35^ This interpretation is supported by the GLMM, which showed between‐farm variance in baseline *Salmonella* positivity, exceeding year‐to‐year variability. Positivity was highest in broilers (5.8%), whereas layers and breeders rarely tested positive. Poultry category was not statistically significant in the GLMM, although layers and breeders showed a non‐significant tendency towards lower odds than broilers. The higher turnover and production intensity of broiler operations may increase exposure to *Salmonella* contamination sources.^36^, ^37^, ^38^ Both vertical and horizontal transmission contribute to *Salmonella* infection.^39^ Vertical transmission can be limited by *Salmonella*‐free chicks, whereas horizontal transmission can be reduced through uncontaminated feed and water, thorough cleaning and disinfection, all‐in/all‐out production where applicable, and appropriate application and implementation of BMs.^40^, ^41^, ^42^, ^43^, ^44^

All hatchery samples were negative. In Serbia, chicks delivered to farms are accompanied by official health certificates confirming origin from *Salmonella*‐free hatcheries, based on regular testing under national regulations.^13^ Introduction may therefore be more likely during transport or after placement on farms than within the hatcheries included in this dataset; however, this inference cannot be confirmed without molecular typing.

In broiler slaughterhouses, *Salmonella* positivity (8.4%) was higher than in farm samples, and repeated detections occurred in the same facilities over several months. Positive results occurred only in meat samples, highlighting the value of this sample type for detecting *Salmonella* contamination.^45^, ^46^, ^47^ Effective *Salmonella* control during processing requires strict hygiene management, including prevention of carcass‐to‐carcass contamination, adequate sanitation of equipment and food‐contact surfaces, and continuous microbiological monitoring.^46^, ^47^, ^48^, ^49^, ^50^ Farm biosecurity and slaughterhouse hygiene interventions should therefore be considered complementary components of an integrated *Salmonella*‐control strategy.

Within the Serbian broiler production chain, a discrepancy was noted between the number of *Salmonella*‐positive samples detected on farms and those detected at slaughterhouses. Although *Salmonella* contamination may increase during processing, molecular typing was not performed, and the origin or direction of contamination could not be established. Moreover, farm samples originated from the Belgrade city epizootiological area, whereas slaughterhouse samples came from facilities in several Serbian provinces that received poultry from diverse geographic sources. This systematic difference in source populations precludes direct comparison between farm and slaughterhouse samples.

Human salmonellosis shows a seasonal pattern, with cases peaking in warmer months, probably because higher ambient temperatures affect food and environmental sources. Secondary winter peaks may reflect seasonal differences in diet, food handling, and care‐seeking behaviour.^9^, ^51^ In poultry, a consistent seasonal pattern has not been established, partly because poultry‐house microclimates are maintained throughout the year. In this study, seasonal differences were observed in both farm and slaughterhouse samples. On farms, the odds of *Salmonella* detection were higher in winter than in spring and summer, consistent with studies from Germany, Spain, Croatia and Poland.^35^, ^52^, ^53^, ^54^ During colder months, rodents seeking shelter in poultry houses and increased condensation in feed mills may contribute to higher *Salmonella* contamination in poultry environments.^35^, ^54^, ^55^ In slaughterhouses, the odds of *Salmonella* detection appeared higher in spring than in winter. Similar seasonal increases in *Salmonella* occurrence have been reported in retail poultry products and slaughterhouse samples in Greece and South Korea.^56^, ^57^

Effective farm biosecurity is crucial for preventing pathogen introduction and spread and protecting animal and human health.^58^, ^59^, ^60^ Although Serbia is among the countries with the largest number of BMs established through legislative and non‐legislative frameworks for different poultry species,^29^ several biosecurity challenges still persist. Survey respondents identified ‘biological vector control’, ‘manure and carcass removal’, ‘depopulation process’ and ‘disease management’ as particularly difficult to implement. These measures often require substantial labour, time and adequate infrastructure, which may explain why they tend to be perceived as more demanding. Several BCs are also directly relevant to *Salmonella* introduction and persistence. Inadequate rodent control is associated with *Salmonella* persistence in poultry holdings,^35^, ^59^ and this factor may contribute to the between‐farm heterogeneity identified by the GLMM. Similarly, deficiencies in manure management and depopulation procedures may promote *Salmonella* environmental persistence and between‐cycle carry‐over,^35^, ^59^ potentially contributing to repeated detections on some farms. The findings also align with previously reported uneven compliance with biosecurity standards on Serbian broiler farms.^60^, ^61^ Similar challenges occur in other livestock sectors, where practical, financial and time constraints, limited institutional support, and perceived impracticality of BMs can outweigh awareness of importance.^21^, ^62^, ^63^, ^64^ Given the relevance of certain *Salmonella* serovars as zoonotic, targeted training, advisory support and context‐specific solutions are needed.^19^

## CONCLUSION

This study documented *Salmonella* spp. occurrence in Serbian poultry production between 2020 and 2025, with *S*. Enteritidis dominant among serotyped farm‐origin isolates. Overall, the findings emphasise the need for robust farm biosecurity and strict slaughterhouse hygiene to reduce *Salmonella* contamination and potential human exposure. Although the retrospective design and partial serotyping limit interpretation, the study provides evidence on recent *Salmonella* occurrence and biosecurity challenges in Serbia. Coordinated action throughout the production chain is important, particularly given reports of antimicrobial‐resistant *Salmonella* strains.^65^, ^66^, ^67^, ^68^ Farmers have a central role in this process, and addressing practical constraints, economic limitations and the perceived impracticality of some BMs is essential for sustainable disease prevention.

## AUTHOR CONTRIBUTIONS

*Conceptualization*: Jelena Maletić, Nemanja Zdravković, Jasna Kureljušić, Branislav Kureljušić, Oliver Radanović, Nemanja Jezimirović and Giulia Graziosi. *Data curation*: Jelena Maletić and Giulia Graziosi. *Formal analysis*: Giulia Graziosi and Marco De Nardi. *Investigation*: Jelena Maletić, Nemanja Zdravković, Jasna Kureljušić, Branislav Kureljušić, Oliver Radanović and Nemanja Jezimirović. *Methodology*: Jelena Maletić, Giulia Graziosi, Branislav Kureljušić, Oliver Radanović and Nemanja Jezimirović. *Resources*: Jelena Maletić, Jasna Kureljušić and Branislav Kureljušić. *Supervision*: Giulia Graziosi. *Validation*: Jelena Maletić and Giulia Graziosi. *Writing—original draft*: Jelena Maletić and Giulia Graziosi. *Writing—review and editing*: Giulia Graziosi and Jelena Maletić. All the authors read and approved the final manuscript.

## CONFLICTS OF INTEREST

The authors declare they have no conflicts of interest.

## ETHICS STATEMENT

This study was conducted in accordance with the ethical principles of Regulation (EU) 2016/679 (General Data Protection Regulation) and the University of Bologna guidelines for the protection of personal data in surveys. Under institutional guidelines for minimal‐risk, non‐interventional research involving adult participants and non‐sensitive data, formal ethical approval was not required. The research was limited to the voluntary collection of opinions and perceptions. Informed consent was obtained from all respondents before they completed the questionnaire. No sensitive personal data were processed or published.

## Supporting information




**Supporting Information 1**. Samples processed as part of routine diagnostic submissions to the Scientific Institute of Veterinary Medicine of Serbia (https://nivs.rs/?lang=en).

Supporting Information

## Data Availability

Some of the data presented in this study are available upon request from the corresponding author. The data are not publicly available due to privacy concerns.
