# Supplementary material for: Salmonella spp. in Serbian poultry production (2020–2025): Retrospective trends and farmers’ perceptions of biosecurity
Source: Vet Rec Open. 2026 Sep 14;13(2):e70049. doi: 10.1002/vro2.70049 (PMC13573095; doi:10.1002/vro2.70049)
Supplement: Supplementary file 1 [file VRO2-13-e70049-s001.docx]

**Supporting Information 2**

**S2.1. Logistic mixed-effects regression model for analysing *Salmonella* occurrence in poultry-farm samples.**

R code used to investigate whether *Salmonella* spp. detection varied by season and poultry type using a generalised linear mixed-effects model (GLMM).

\# Generalised linear mixed-effects model

model1 <- glmmTMB(Results ~ Type_of_poultry + Season + (1 | Farm_ID) + (1 | Year), data = data, family = binomial(link = "logit"))

Results = presence or absence of *Salmonella* in each farm sample

Type_of_poultry = poultry category (broilers, layers, and breeder flocks)

Season = winter, spring, summer, or autumn

data = dataset of *Salmonella* monitoring on poultry farms

#Model diagnostics

\## Check for overdispersion and zero inflation

simulationOutput <- simulateResiduals(model1)

plot(simulationOutput)

testDispersion(simulationOutput)

testZeroInflation(simulationOutput)

\## Model performance and multicollinearity

performance::check_model(model1)

performance::r2(model1)

check_collinearity(model1)

Figure S1. Non-parametric dispersion test comparing the standard deviation of fitted residuals with simulated residuals. The plot indicates adequate model fit.

Figure S2. Quantile-quantile plot (QQ plot) of residuals (left) and residuals versus predicted values (right). No major distributional problems or residual patterns are apparent.

Figure S3. Zero-inflation test. There was no evidence of excess zeros.

**S2.2. Logistic mixed-effects regression model for analysing *Salmonella* occurrence in broiler slaughterhouse samples**

R code used to investigate whether *Salmonella* spp. detection varied by season using a generalised linear mixed-effects model (GLMM).

\# Generalised linear mixed-effects model

Model2 <- glmmTMB(Results ~ Season + (1 | Slaughterhouse_ID) + (1 | Year), data = data, family = binomial(link = "logit"))

Results = presence or absence of *Salmonella* in meat samples

Season = winter, spring, summer, or autumn

data = dataset of *Salmonella* monitoring in broiler slaughterhouses

#Model diagnostics

\## Check for overdispersion and zero inflation

simulationOutput <- simulateResiduals(model2)

plot(simulationOutput)

testDispersion(simulationOutput)

testZeroInflation(simulationOutput)

\## Model performance and multicollinearity

performance::check_model(model2)

performance::r2(model2)

check_collinearity(model2)

Figure S4. Non-parametric dispersion test comparing the standard deviation of fitted residuals with simulated residuals. The plot indicates adequate model fit.

Figure S5. Quantile-quantile plot (QQ plot) of residuals (left) and residuals versus predicted values (right). No major distributional problems or residual patterns are apparent.

Figure S6. Zero-inflation test. There was no evidence of excess zeros.
